# AMP‐activated protein kinase inhibition in fibro‐adipogenic progenitors impairs muscle regeneration and increases fibrosis

**DOI:** 10.1002/jcsm.13150

**Published:** 2022-12-13

**Authors:** Xiangdong Liu, Liang Zhao, Yao Gao, Yanting Chen, Qiyu Tian, Jun Seok Son, Song Ah Chae, Jeanene Marie de Avila, Mei‐Jun Zhu, Min Du

**Affiliations:** ^1^ Laboratory of Nutrigenomics and Growth Biology, Department of Animal Sciences Washington State University Pullman Washington USA; ^2^ College of Animal Science and Technology Nanjing Agricultural University Nanjing PR China; ^3^ School of Food Science Washington State University Pullman Washington USA

**Keywords:** AMPK, FAPs, fibrosis, MMP‐9, obesity, TGF‐β

## Abstract

**Background:**

Following muscle injury, fibro‐adipogenic progenitors (FAPs) are rapidly activated and undergo apoptosis at the resolution stage, which is required for proper muscle regeneration. When excessive FAPs remain, it contributes to fibrotic and fatty infiltration, impairing muscle recovery. Mechanisms controlling FAP apoptosis remain poorly defined. We hypothesized that AMP‐activated protein kinase (AMPK) in FAPs mediates their apoptosis during the muscle regeneration.

**Methods:**

To test, AMPKα1^fl/fl^ PDGFRα^Cre^ mice were used to knock out AMPKα1 in FAPs. Following AMPKα1 knockout, the mice were injected with phosphate‐buffered saline or glycerol to induce muscle injury. Tibialis anterior muscle and FAPs were collected at 3, 7 and 14 days post‐injury (dpi) for further analysis.

**Results:**

We found that AMPKα1 deletion in FAPs enhanced p65 translocation to the nuclei by 110% (*n* = 3; *P* < 0.01). AMPKα1 knockout group had a higher gene expression of MMP‐9 (matrix metalloproteinase‐9) by 470% (*n* = 3; *P* < 0.05) and protein level by 39% (*n* = 3; *P* < 0.05). Loss of AMPKα1 up‐regulated the active TGF‐β1 (transforming growth factor‐β1) levels by 21% (*n* = 3; *P* < 0.05). TGF‐β promoted apoptotic resistance, because AMPKα1‐deficient group had 36% lower cleaved Caspase 3 (cCAS3) content (*n* = 3; *P* < 0.05). Fibrotic differentiation of FAPs was promoted, with increased collagen protein level by 54% (*n* = 3; *P* < 0.05). Moreover, obesity decreased phosphorylation of AMPK by 54% (*n* = 3; *P* < 0.05), which decreased cCAS3 in FAPs by 44% (*n* = 3; *P* < 0.05) and elevated collagen accumulation (52%; *n* = 3; *P* < 0.05) during muscle regeneration.

**Conclusions:**

These data suggest that AMPK is a key mediator of FAPs apoptosis, and its inhibition due to obesity results in fibrosis of regenerated muscle.

## Introduction

As the most abundant tissue, skeletal muscle has numerous functions, including energy metabolism, movement, protection of organs and endocrine functions.^1^, ^2^ Skeletal muscle possesses a robust capacity of repair and regeneration in response to injury caused by exercise or trauma.^3^, ^4^ Muscle regeneration is tightly regulated, and improper regeneration results in the replacement of muscle fibres by fibrotic and fatty tissues, progressively worsening muscle functions.^5^, ^6^ Following injury, several key cell populations interact with each other to coordinate muscle regeneration, including satellite cells, fibro‐adipogenic progenitors (FAPs) and macrophages.^7^

Shortly after injury, FAPs are rapidly activated, which stimulate satellite proliferation.^8^ FAPs secrete multiple molecules, including interleukin (IL)‐6, IL‐10 and Wnt family member 1 (WNT1) inducible signalling pathway protein 1 (WISP1), which regulate the proliferation and differentiation of satellite cells.^9^, ^10^, ^11^, ^12^ In addition, FAPs secrete IL‐33, which recruits regulatory T cells (Tregs).^13^ Tregs secrete IL‐10 to suppress inflammation, aiding muscle recovery after injury.^14^ FAPs proliferate and peak at 2–3 days after injury. After this transient expansion, FAPs undergo apoptosis and phagocytic clearance.^9^, ^10^ However, under chronic inflammation and muscular dystrophies, failure of FAPs clearance may occur, and excessive FAPs further differentiate into adipocytes and fibroblasts, causing fibrotic and fatty infiltration and impairing the function of regenerated muscle.^11^, ^15^, ^16^

AMP‐activated protein kinase (AMPK) is a nutrient and energy sensor, serving pivotal roles in maintaining energy homeostasis.^17^ Notably, AMPK also regulates skeletal muscle development and regeneration.^18^ During muscle regeneration, inflammatory response is meticulously modulated.^19^ AMPK has potent anti‐inflammatory effects.^20^ AMPK activation inhibits nuclear factor‐κB (NF‐κB) signalling, a canonical inflammatory signalling pathway, which regulates the expression of multiple pro‐inflammatory genes and activation of immune cells.^21^, ^22^ Additionally, macrophages are involved in all phases of tissue repair. During the initial stage of muscle regeneration, macrophages are in a M1 inflammatory state, which transits to M2 anti‐inflammatory state at the regeneration stage.^23^ AMPKα1 is required for phenotypic transition of macrophages, facilitating muscle recovery. Deletion of AMPKα1 in macrophages blocks their M2 transition and muscle regeneration.^24^ Moreover, AMPK regulates the homeostasis of satellite cell populations. Suppression of AMPK activity in obese mice attenuates satellite cell activity and muscle regeneration.^25^ However, the role of AMPK in regulating the proliferation and subsequent apoptosis of FAPs during muscle regeneration remains unexamined.

TGF‐β (transforming growth factor‐β) is a master regulator of tissue fibrosis.^26^ The canonical pathway, TGF‐β/SMADs, triggers the transcription of several profibrotic genes, including *Atat2*, *collagen I* and *tissue inhibitor of matrix metalloproteinases* (*TIMP*).^27^, ^28^ In addition, TGF‐β induces the differentiation of FAPs into myofibroblasts,^29^ which are the main producers of extracellular matrix (ECM).^30^

AMPK activation suppresses TGF‐β.^31^, ^32^, ^33^ However, the role of AMPK–TGF‐β axis on FAP cellular changes during muscle regeneration remains unexamined. Thus, it is important to define the roles of AMPK/TGF‐β axis in FAP apoptosis and their subsequent production of fibrotic tissue during muscle regeneration.

## Methods

### Animals

All animal procedures were conducted according to the protocol approved by the Institute of Animal Use and Care Committee (IAUCC) at Washington State University. C57B/6J male (WT) mice at 10 weeks were fed with either a control diet (ND; 10% energy from fat, D12450; Research Diets, New Brunswick, NJ, USA) or high‐fat diet (HFD; 60% energy from fat, D12492; Research Diets) for 12 weeks.

To produce *Pdgfrα‐creER™* ^*(−/+)*^ */Prkaa1* ^*fl/fl*^ mice, *B6N.Cg‐Tg (Pdgfra‐cre/ERT) 467Dbe/J* (*Pdgfra‐creER™*, Stock No. 018280, Jackson Lab, Bar Harbor, ME, USA) were crossed with *Prkaa1* ^*tm1.1Sjm*^/J (*Prkaa1* ^*fl*^, Stock No. 014141, Jackson Lab). To obtain mice with conditional AMPKα1 whole‐body knockout (KO) mice, B6;129‐*Gt (ROSA)26Sor* ^*tm1(cre/ERT)Nat*^/J mice (*R26CreER*, Stock No. 004847, Jackson Lab) were bred with *Prkaa1* ^*fl*^ mice. All mice were housed in an environmentally controlled condition (23 ± 2°C, 12–12 h light–dark cycle). At the age of 2 months, the male mice with similar body weight were injected with tamoxifen intraperitoneally at the concentration of 75 mg/kg (tamoxifen/body weight) for 3 continuous days. Two days after the last injection, tibialis anterior (TA) muscle was intramuscularly injected with 50 μL 50% glycerol (glycerol/phosphate‐buffered saline [PBS]) to induce muscle injury. TA muscles were collected at 3, 7 and 14 days post‐injury (dpi).

### Muscle histological examination

TA muscle was fixed in fresh 4% paraformaldehyde (PFA) and cryopreserved in 30% sucrose. Obtained samples were embedded in optimal cutting temperature (OCT) compound (#23730571, Fisher Scientific, Houston, TX, USA) in precooled isopentane in liquid nitrogen. Sections were obtained and used for haematoxylin and eosin (H&E) or Masson trichrome staining. For immunofluorescence staining, sections were heated in citrate buffer (pH = 6) for 20 min and blocked with 1% bovine serum albumin (BSA) in Tris‐buffered saline (TBS) containing 0.3% Triton X100 for 1 h. Sections were incubated with primary antibodies overnight in 4°C as follows: anti‐COL1a (#59772, RRID:AB_1121,787; Santa Cruz, Dallas, TX, USA); anti‐PDGFRa (#AF1062, RRID:AB_2236,897; R&D Systems, Minneapolis, MN, USA) and anti‐cleaved Caspase 3 (#9664, RRID:AB_2070,042; Cell Signaling Technology, Danvers, MA, USA). After washing, the sections were incubated with the corresponding fluorescent secondary antibodies for 1 h. The sections were washed and mounted in a mounting medium with DAPI (#H‐1500, Vector Laboratories, Burlingame, CA, USA). Immunofluorescence imaging was conducted using a fluorescence microscope (EVOS FL, Life Technologies, Carlsbad, CA, USA).^34^ ImageJ software (National Institutes of Health [NIH]) was used to measure the collagen area following Masson trichrome staining, the size distribution of regenerated myofibers in H&E staining and the ratio of COL1α+ areas in regenerated TA muscle by fluorescence staining. FAP cell numbers were calculated by assessing the number of PDGFRα+ cells with nuclei identified by DAPI staining in each field. For every animal or cell samples, four representative sections or fields were used for measurements.^34^

### Primary fibro‐adipogenic progenitor isolation and purification

FAPs were isolated as previously described.^35^, ^36^ Briefly, lower limb muscles were collected, finely minced and digested in 800 U/mL Collagenase II (#17101015, Gibco, Grand Island, NY, USA) in Dulbecco's modified Eagle's medium (DMEM; #10313021, Gibco, Grand Island, NY, USA) for 1 h at 37°C. After washing, the muscle slurries were further digested in 100 U/mL Collagenase II and 1.1 U/mL Dispase II (#17105041, Gibco, Grand Island, NY, USA) for 30 min. Then, the obtained muscle slurries were filtered through 100 mm (#22363549, Fisher Scientific) and 40 mm (#22363547, Fisher Scientific) cell strainer. Filtered slurries were centrifuged at 400 g. The pellets were resuspended in magnetic‐activated cell sorting (MACS) buffer consisting of PBS with 2% fetal bovine serum (FBS) and 2 mM EDTA, followed by incubation with anti‐CD16/32 antibody (#101302, RRID:AB_312801; BioLegend, San Diego, CA, USA) for 5 min to block nonspecific binding. To isolate cells magnetically, cells were incubated with biotinylated antibodies against anti‐a7 integrin (#A130501979, Miltenyi Biotec, San Jose, CA, USA), anti‐CD45 (#109804, RRID:AB_313441; BioLegend) and anti‐CD31 (#102404, RRID:AB_312,899; BioLegend) for 15 min at 4°C. After that, cells were washed with MACS buffer and incubated with anti‐biotin microbeads (#120000900, Miltenyi Biotec). Subsequently, the obtained mixture was loaded on LD columns (#130042901, Miltenyi Biotec). The flow through fraction was collected and incubated with an anti‐SCA1‐PE antibody (#108108, RRID:AB_313345; BioLegend) for 15 min at 4°C and then with anti‐PE microbeads (#130048801, Miltenyi Biotec). The retained cells were collected.

### Cell culture

Newly isolated FAPs (P0) were cultured in DMEM, consisting of 20% heat‐inactivated FBS (#10439001, Gibco, Carlsbad, CA, USA), 1% penicillin–streptomycin (#P0781, Sigma, St. Louis, MO, USA) and 2.5 ng/mL of basic fibroblast growth factor (bFGF) (#PHG0021, Gibco, Carlsbad, CA, USA). FAPs at Passages 2 or 3 were used for experiments. For spontaneous differentiation, FAPs were cultured in DMEM, consisting of 10% heat‐inactivated FBS, 1% penicillin–streptomycin for 7 days followed by fibrotic evaluation. For apoptosis induction, FAPs were treated with DMEM containing 10% FBS, 1% penicillin–streptomycin and 100 ng/mL tumour necrosis factor‐α (TNF‐α) (#575202, BioLegend) for 48 h. Moreover, FAPs were transferred with green fluorescent protein (GFP) (13031, Addgene), wild‐type AMPK (WT, 15911, Addgene) or K45R mutant AMPK (K45R, 15992, Addgene). Obtained cells were cultured in serum‐free DMEM/F12 media supplemented with 2 ng/mL latent TGF‐β (299‐LT‐005/CF, R&D Systems) for 1 day.

### Immunocytochemical staining

Cells were fixed in chilled methanol on ice for 10 min. After washing with PBS, cells were treated with 0.1% Triton X‐100 at room temperature (RT) for 5 min and blocked with 2% BSA. Sequentially, cells were incubated with primary antibodies including cleaved Caspase 3 (#9664, RRID:AB_2070042, Cell Signaling Technology) or COL1α (#59772, RRID:AB_1121787, Santa Cruz) at 4°C overnight. After washing, cells were incubated with corresponding secondary antibodies at RT for 1 h. Cells were then washed and mounted in a mounting medium with DAPI (#H‐1500, Vector Laboratories). Immunofluorescence images were taken using a fluorescence microscope (EVOS FL).

### Quantitative real‐time PCR analyses

Total RNA was isolated with Trizol reagent (#T9424, Sigma) and followed by deoxyribonuclease treatment to degrade DNA. The cDNA templates were synthesized using an iScript cDNA synthesis kit (#1708891, Bio‐Rad, Hercules, CA, USA). A SYBR Green RT‐PCR kit (#1725274, Bio‐Rad) was used to conduct quantitative real‐time PCR (qRT‐PCR) with the CFX Connect™ Real‐Time PCR detection system (Bio‐Rad). 18S RNA was used as a reference gene to normalize mRNA expression levels. 2^−ΔΔCt^ method was used to analyse relative mRNA expression.^37^ Primer sequences are listed in *Table* *1*.

**TABLE 1:** Primer sequences used for real‐time quantitative PCR

Gene	Forward (5′‐3′)	Reverse (5′‐3′)	Size (bp)	Access no.
*Acta2*	ATGCTCCCAGGGCTGTTTTCCCAT	GTGGTGCCAGATCTTTTCCATGTCG	191	NM_007392.3
*Bmp1*	CACTGTGTATGGCGCATCTC	TAGTCATACCAGCACAGGCG	98	NM_001360021.1
*Col1a*	GCTCCTCTTAGGGGCCACT	CCACGTCTCACCATTGGGG	103	NM_007742.4
*Col3a*	CTGTAACATGGAAACTGGGGAAA	CCATAGCTGAACTGAAAACCACC	144	NM_009930.2
*Cebpa*	CAAGAACAGCAACGAGTACCG	GTCACTGGTCAACTCCAGCAC	124	NM_001287514.1
*Eda‐fibronectin*	GATGGTGAAGACGACACTGC	GAATGGCTGTGGACTGGATT	127	NM_010233.2
*Fabp4*	CGACAGGAAGGTGAAGAGCATCATA	CATAAACTCTTGTGGAAGTCACGCCT	158	NM 024406.2
*Fibronectin*	ATGTGGACCCCTCCTGATAGT	GCCCAGTGATTTCAGCAAAGG	124	NM_010233.2
*Furin*	CATGACTACTCTGCTGATGG	GAACGAGAGTGAACTTGGTC	148	X54056.1
*Ifng*	AGCAAGGCGAAAAAGGATGC	TCATTGAATGCTTGGCGCTG	83	NM_008337.4
*Il1b*	TCGCTCAGGGTCACAAGAAA	CATCAGAGGCAAGGAGGAAAAC	73	XM_006498795.3
*Il6*	GAGGATACCACTCCCAACAGACC	AAGTGCATCATCGTTGTTCATACA	141	NM_001314054.1
*Il22*	TGCGATCTCTGATGGCTGTC	GACGATGTATGGCTGCTGGA	170	NM_016971.2
*Mmp‐2*	CATGCGGAAGCCAAGATGTG	GTTTCAGGGTCCAGGTCAGG	129	NM_008610.3
*Mmp‐9*	TGAATCAGCTGGCTTTTGTG	GTGGATAGCTCGGTGGTGTT	242	NM_013599.5
*Mmp‐9* (ChIP)	GCGGACATTGTCATCCAGTTTG	CGTCGTCGAAATGGGCATC	130	NM_013599.5
*Mmp‐14*	TCACCCCAGCATTGCTTCAT	CACACACCGAGCTGTGAGAT	99	NM_008608.4
*Myf5*	AAACTCCGGGAGCTCCGCCT	GGCAGCCGTCCGTCATGTCC	125	NM_008656.5
*Myod*	TCTGGAGCCCTCCTGGCACC	CGGGAAGGGGGAGAGTGGGG	100	NM_010866.2
*Myogenin*	GAGATCCTGCGCAGCGCCAT	CCCCGCCTCTGTAGCGGAGA	97	NM_031189.2
*Pparg*	AGCTCCAAGAATACCAAAGTGCGAT	AGGTTCTTCATGAGGCCTGTTGTAGA	98	XM 017321456.1
*Tcf4*	GGCGATGAGAACCTGCAAGA	GGTCCTCATCATCGTTATTGCTAGA	115	NM_013685.2
*Thbs1*	GCTATCTGTGGCCTCTCCTG	TTCAGCTCACTGACCAGCTC	137	BC050917.1
*Tnfa*	TGGGACAGTGACCTGGACTGT	TTCGGAAAGCCCATTTGAGT	67	NM_001278601.1
*Tgfb1*	ACTGGAGTTGTACGGCAGTG	GGGGCTGATCCCGTTGATTT	123	NM_011577.2

### Immunoblotting analysis

**Figure 1:**
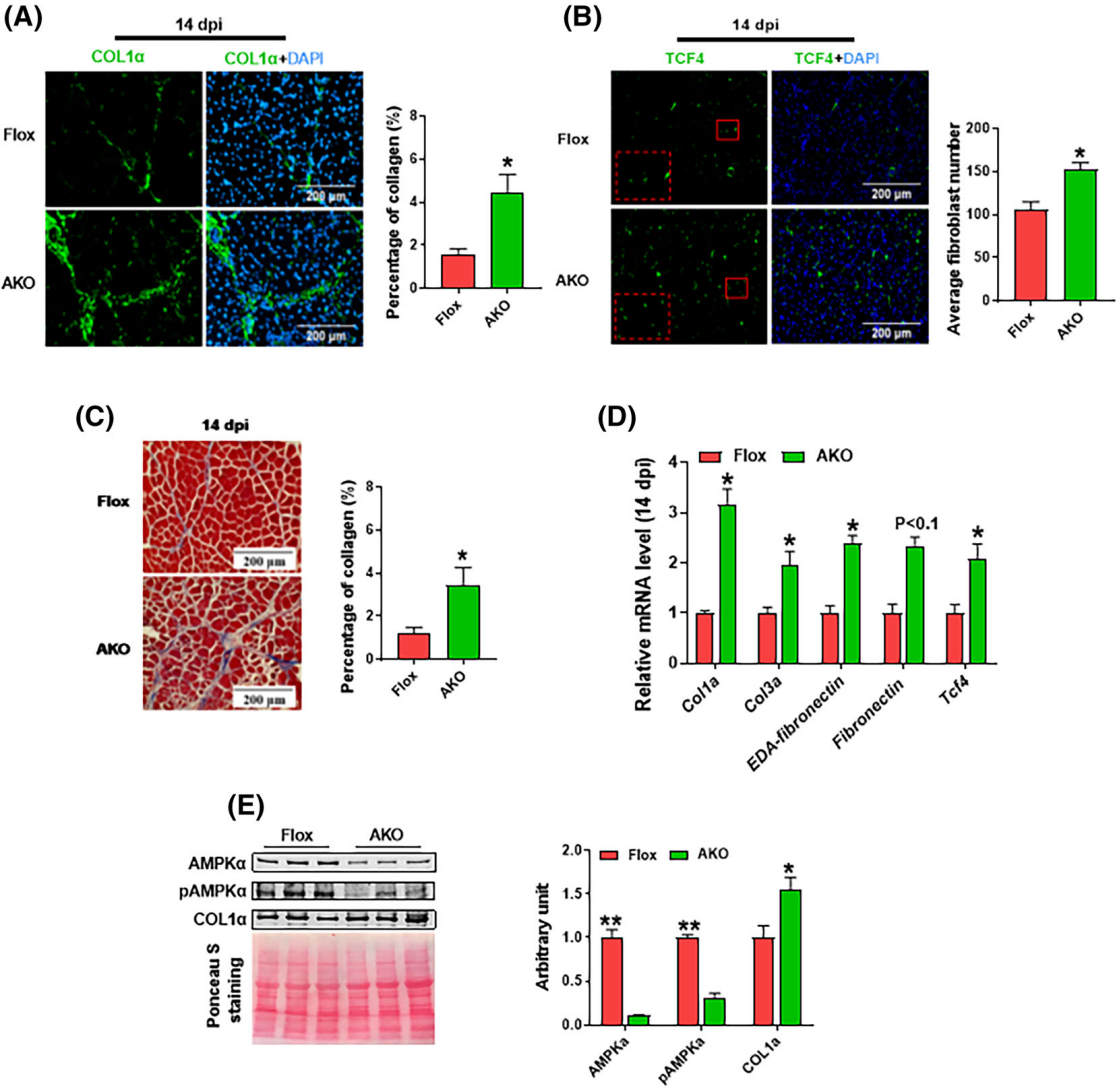
AMPKα1 knockout promotes fibrosis in regenerated muscle tissue. (A) Immunofluorescence staining of COL1α and the percentage of COL1α+ areas in regenerated muscle at 14 days post‐injury (dpi). (B) Immunofluorescence staining of TCF4 and the percentage of TCF4+ cells in regenerated muscle at 14 dpi. (C) Masson trichrome staining of regenerated muscle at 14 dpi and the percentage of connective tissue areas per field. (D) Relative mRNA expression of fibrotic markers including *Col1a*, *Col3a*, *EDA‐fibronectin*, *Fibronectin* and *Tcf4* in regenerated muscle at 14 dpi. (E) Western blotting analysis of AMPKα, phospho (p)‐AMPKα and COL1α in regenerated muscle at 14 dpi. Data are shown as the means ± SEM (*n* = 3). *P < 0.05, **P < 0.01. Bars, 200 μm

Immunoblotting analyses were conducted as previously described using the Odyssey Infrared Image System (LICOR Biosciences, Lincoln, NE, USA).^34^ Western blot for AMPK KO muscle tissue was normalized by the total protein using Ponceau S staining, including *Figures* *1* *E* and *4* *C,D*. p65 in *Figure* *5* *C* was normalized by Histone 3. For the others, TUBULIN was used as a housekeeping protein. The primary antibodies used were anti‐AMPKα (#2532S), anti‐phospho‐AMPKα (#2535S), anti‐SMAD3 (#9513S), anti‐Phospho‐SMAD3 (#9520S), anti‐TGF‐β (#3711), anti‐MMP‐9 (#3852), anti‐p65 (#8242), β‐tubulin (#2146S) and anti‐COL1α antibody (#59772). All antibodies were purchased from Cell Signaling Technology, except for anti‐COL1α antibody (#59772) that was purchased from Santa Cruz Biotech.

### Subcellular fractionation of fibro‐adipogenic progenitors

Isolation of nuclei and cytosol of FAPs was conducted according to a previous report.^38^ Briefly, cells were scraped and washed with cold PBS. Obtained cells were resuspended in 300–500 μL buffer consisting of 50 mM Tris–HCl (pH = 7.4), 250 mM sucrose, 5 mM MgCl_2_ and a protein inhibitor cocktail (#P2714, Sigma) and homogenized with a pellet pestle. The homogenate was maintained on ice for 30 min, followed by vortexing for 15 s at maximum speed and then centrifuged at 800 g for 15 min. The obtained pellet was washed by resuspending in 500 μL of the same buffer, vortexed and centrifuged at 500 g for 15 min twice.

The pellet was resuspended in 200–500 μL buffer consisting of 20 mM HEPES (pH = 7.9), 0.5 M NaCl, 0.2 mM EDTA, 1.5 mM MgCl_2_, 20% glycerol, 1% Triton‐X‐100 and a protein inhibitor cocktail and then homogenized and incubated on ice for 30 min. Subsequently, this fraction was sonicated and centrifuged at 9000 g for 30 min at 4°C, and the supernatant was used for protein extraction.

### Chromatin immunoprecipitation quantitative PCR

Chromatin immunoprecipitation quantitative PCR (ChIP‐qPCR) was performed according to a previous report with some adjustments.^39^ Briefly, harvested cells were fixed followed by glycine treatment. Collected cells were washed and treated with a lysis buffer supplemented with a protein inhibitor cocktail (#P2714, Sigma). Then, the mixture underwent sonication to produce 200–400 bp chromatin segments. After centrifugation, the obtained supernatant was pre‐blocked with ChIP‐grade Protein G Magnetic Beads (#9006, Cell Signaling Technology) and incubated with anti‐p65 antibody (#8242, Cell Signaling Technology) and regular rabbit IgG overnight at 4°C. The mixture was incubated with ChIP‐grade Protein G Magnetic Beads (#9006, Cell Signaling Technology) for 2 h at 4°C. The magnetic beads were collected and washed. The collected beads were dissolved in elution buffer and treated with NaCl, RNase A and proteinase K. The same volume of mixture of Phenol:Chloroform:Isoamyl alcohol (25:24:1) (#A0417977, ACROS, Fair Lawn, NJ, USA) was added, vortexed and incubated for 20 min at RT. The supernatant was collected. A double volume of ethanol was added to the supernatant and placed at −20°C overnight. Finally, the DNA was extracted. Obtained DNA was used for qRT‐PCR. The primers are listed in *Table* *1*.

### Enzyme‐linked immunosorbent assays

The TGF‐β1 levels in muscle tissue, cell culture supernatant and cells were examined using a mouse TGF‐β1 enzyme‐linked immunosorbent assay (ELISA) kit (EK0515, Boster, Wuhan, China). The experiments were carried out according to the manufacturer's instructions.

### siRNA

Cells were grown in 12‐well plates. Lipofectamine™ 3000 was used to transfect the siRNA based on the manufacturer's instructions (Lipofectamine™ 3000, Carlsbad, CA, USA). Mouse Mmp‐9 siRNA was purchased from Life Technology Corporation (AM16708, Carlsbad, CA, USA).

### Statistics

All data are presented as means ± SEM and analysed by GraphPad Prism 8 (San Diego, CA, USA). A Student's *t*‐test was used to compare two treatments. One‐way ANOVA and post hoc (Tukey's test) analysis were performed when there were more than two groups. Unless indicated specifically, animal and cell experiments had three biological replicates. *P* < 0.05 was considered statistically significant.

## Results

### AMPKα1 knockout in fibro‐adipogenic progenitors promotes fibrosis in regenerated muscle tissue

Several studies have reported that AMPK activation inhibits fibrosis.^33^, ^40^ ^, S1^ We found that AMPKα1 KO in FAPs increased collagen accumulation in the TA muscle at 14 dpi (*Figure* *1* *A*). TCF4 is a marker of fibroblasts.^S2^ Immunofluorescence staining against TCF4 showed that AMPKα1 KO enhanced fibroblast density (*Figure* *1* *B*). Based on Masson trichrome staining, the AMPKα1 KO group had higher collagen content than the control group at 14 dpi (*Figure* *1* *C*). We also analysed the expression of fibrogenic markers at 7 (Supporting Information, *Figure* S1 *A*) and 14 dpi (*Figure* *1* *D*). We found that AMPKα1 KO elevated mRNA expression of fibrosis markers, including *Col1a*, *Col3a* and *Tcf4*. The mRNA level of downstream targets of TGF‐β was also increased by AMPK deletion at 14 dpi (*Figure* *1* *D*). In agreement, the contents of AMPKα and phospho‐AMPKα were substantially lower in regenerated muscle of FAP‐specific AMPK α1 KO mice at 14 dpi, which was associated with a higher content of collagen 1α (*Figure* *1* *E*). These data showed that AMPKα1 KO in FAPs enhanced fibrosis in regenerated muscle.

### Loss of AMPKα1 in fibro‐adipogenic progenitors attenuates muscle fibre regeneration

**Figure 2:**
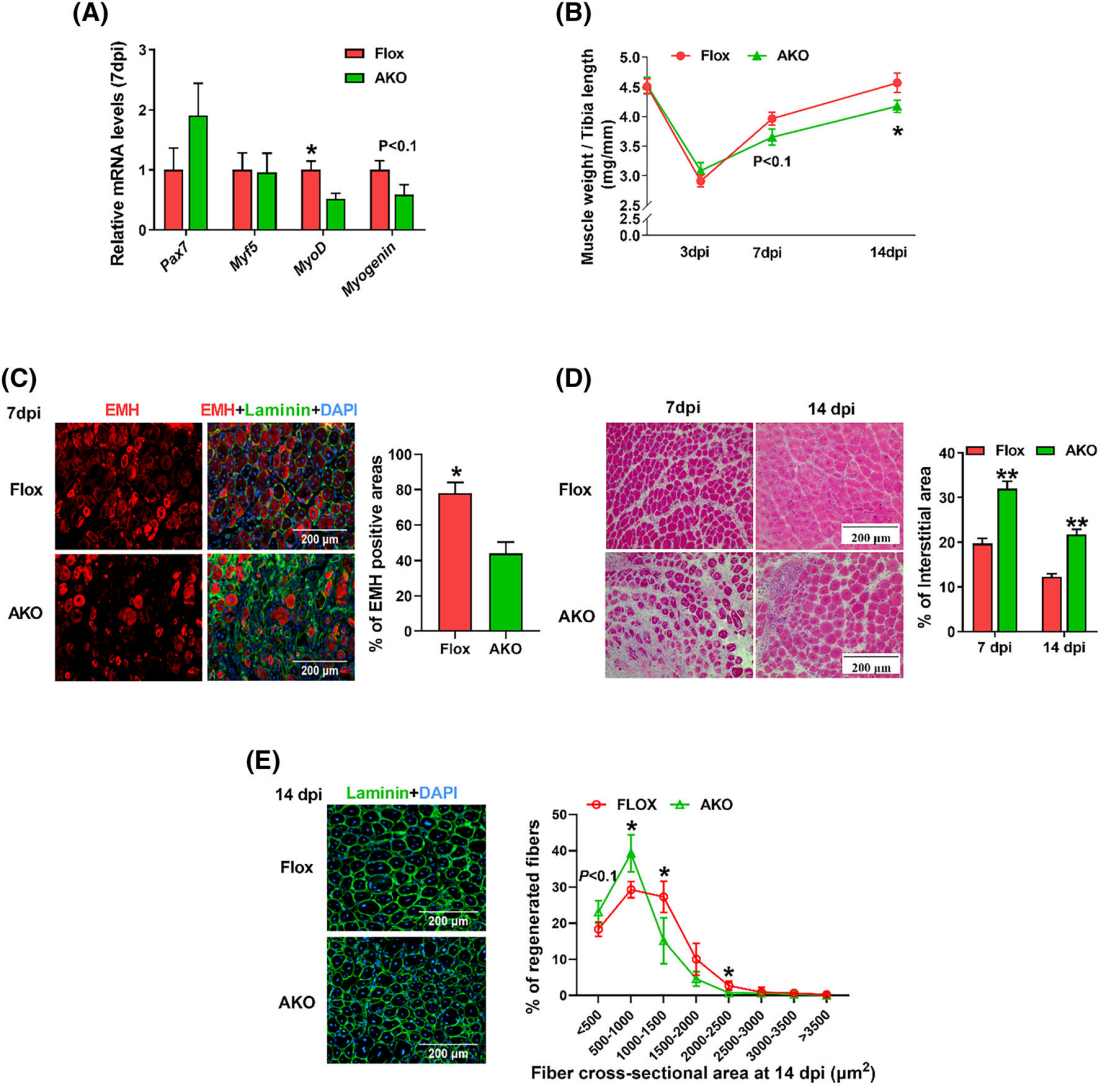
Loss of AMPKα1 in fibro‐adipogenic progenitors attenuates muscle fibre regeneration. (A) Relative mRNA expression of myogenic genes including *Pax7*, *Myf5*, *Myod* and *Myogenin* at 7 days post‐injury (dpi). (B) The weight of regenerated tibialis anterior (TA) muscle normalized to the length at 3, 7 and 14 dpi. (C) Immunofluorescence staining of embryonic myosin heavy (EMH)+ muscle fibres in TA muscle at 7 dpi and quantification. (D) Haematoxylin and eosin (H&E) staining of regenerated skeletal muscle and the percentage of interstitial areas between regenerated muscle fibres. (E) Immunofluorescence staining of laminin and the distribution of cross‐sectional areas of regenerated myofibers. Data are shown as the means ± SEM (*n* = 3). *P < 0.05, **P < 0.01. Bars, 200 μm

During muscle regeneration, FAPs support satellite cell activation and myogenic differentiation.^8^ We further analysed the effects of AMPKα1 KO in FAPs on myogenesis. The mRNA transcription of *MyoD* decreased and *Myogenin* expression tended to decrease due to AMPKα1 KO (*Figure* *2* *A*). The ratio of muscle weight to tibia bone length tended to reduce at 7 dpi and significantly decreased at 14 dpi in AMPKα1 KO mice (*Figure* *2* *B*). Newly regenerated muscle fibres express embryonic myosin heavy (EMH) chain.^S3, S4^ Immunofluorescent staining showed that AMPKα1 ablation reduced EMH expression at 7 dpi (*Figure* *2* *C*). We further measured the sizes of regenerated muscle fibres and interstitial area by H&E staining and immunofluorescence staining of laminin. AMPKα1 KO increased interstitial area at 7 and 14 dpi whereas reduced regenerated muscle fibre sizes at 14 dpi (*Figure* *2* *D,E*). In summary, AMPKα1 deletion in FAPs impaired muscle regeneration.

### AMPKα1 knockout enhances fibrogenic commitment of fibro‐adipogenic progenitors

**Figure 3:**
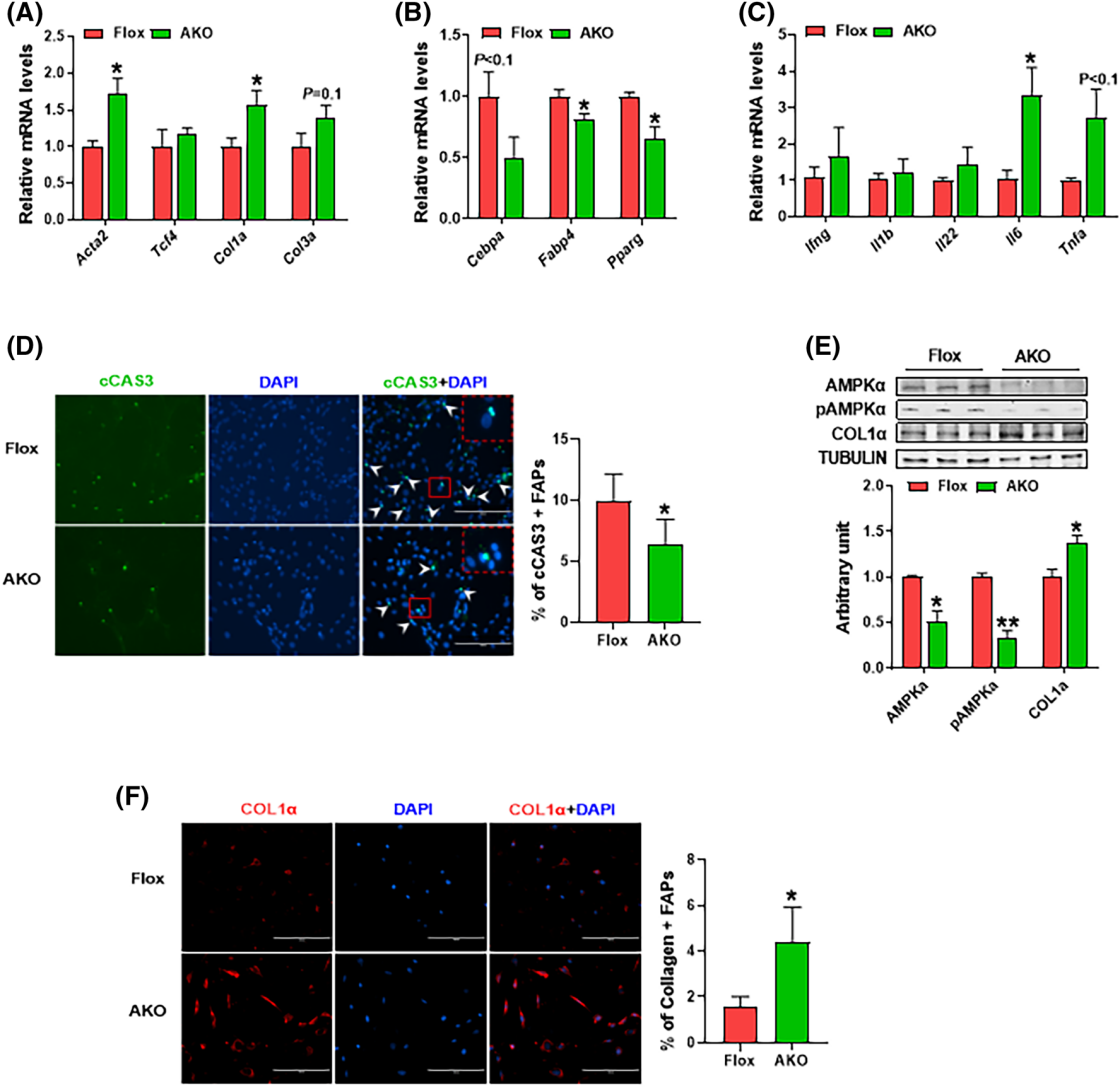
AMPKα1 knockout enhances fibrogenic commitment of fibro‐adipogenic progenitors (FAPs). (A) Relative mRNA expression of fibrotic markers including *Acta2*, *Tcf4*, *Col1a* and *Col3a* in FAPs. (B) Relative mRNA expression of adipogenic markers including *Cebpa*, *Fabp4* and *Pparg* in FAPs. (C) Relative mRNA expression of inflammatory markers including *Ifng*, *Il1b*, *Il22*, *Il6* and *Tnfa* in FAPs. (D) Immunofluorescence analysis of cleaved Caspase 3 (cCAS3)+ FAPs and quantifications of the percentage of cCAS3+ FAPs after 100 ng/mL tumour necrosis factor‐α (TNF‐α) treatment for 48 h. (E) Western blotting analysis of AMPKα, phospho (p)‐AMPKα and COL1α in FAPs cultured in Dulbecco's modified Eagle's medium (DMEM) for 10 days. (F) Immunofluorescence analysis of COL1α and quantifications of the percentage of COL1α+ FAPs after FAPs were cultured in DMEM for 10 days. Data are shown as the means ± SEM (*n* = 3). *P < 0.05, **P < 0.01. Bars, 200 mm

We analysed the expression of fibrotic, adipogenic and inflammatory markers in freshly isolated FAPs. We found that AMPKα1 KO enhanced fibrogenic gene expression, including *Acta2* and *Col1*a (*Figure* *3* *A*). The expression of adipogenic genes *Peroxisome proliferator‐activated receptor gamma* (*Pparg*) and *Fatty‐acid‐binding protein 4* (*Fabp4*) was attenuated due to AMPKα1 deletion. The *CCAAT‐enhancer‐binding protein alpha* (*Cebpa*) expression tended to be down‐regulated in the AMPKα1 KO group (*P* < 0.1) (*Figure* *3* *B*). Moreover, AMPKα1 KO increased pro‐inflammatory marker, *Il6*, and tended to increase the expression of tumour necrosis factor alpha (*Tnfa*) (*Figure* *3* *C*). To measure apoptosis resistance, FAPs were treated with 100 ng/mL TNF‐α for 48 h and followed by immunofluorescence staining. AMPKα1 KO group had less cCAS3+ FAPs than the control group (*Figure* *3* *D*). Consistently, the western blotting data showed that AMPK KO group had higher amount of procaspase 3 and lower level of cleaved Caspase 3 in FAPs (*Figure* *S1* *C*). Additionally, after 7 days of spontaneous differentiation, collagen 1α protein content was enhanced in AMPKα1 KO group, as shown by western blotting and immunofluorescence staining (*Figure* *3* *E,F*). Conclusively, FAPs acquired fibrotic characters due to AMPKα1 KO.

### AMPKα1 knockout enhances transforming growth factor‐β signalling, which is associated with elevated fibrosis

**Figure 4:**
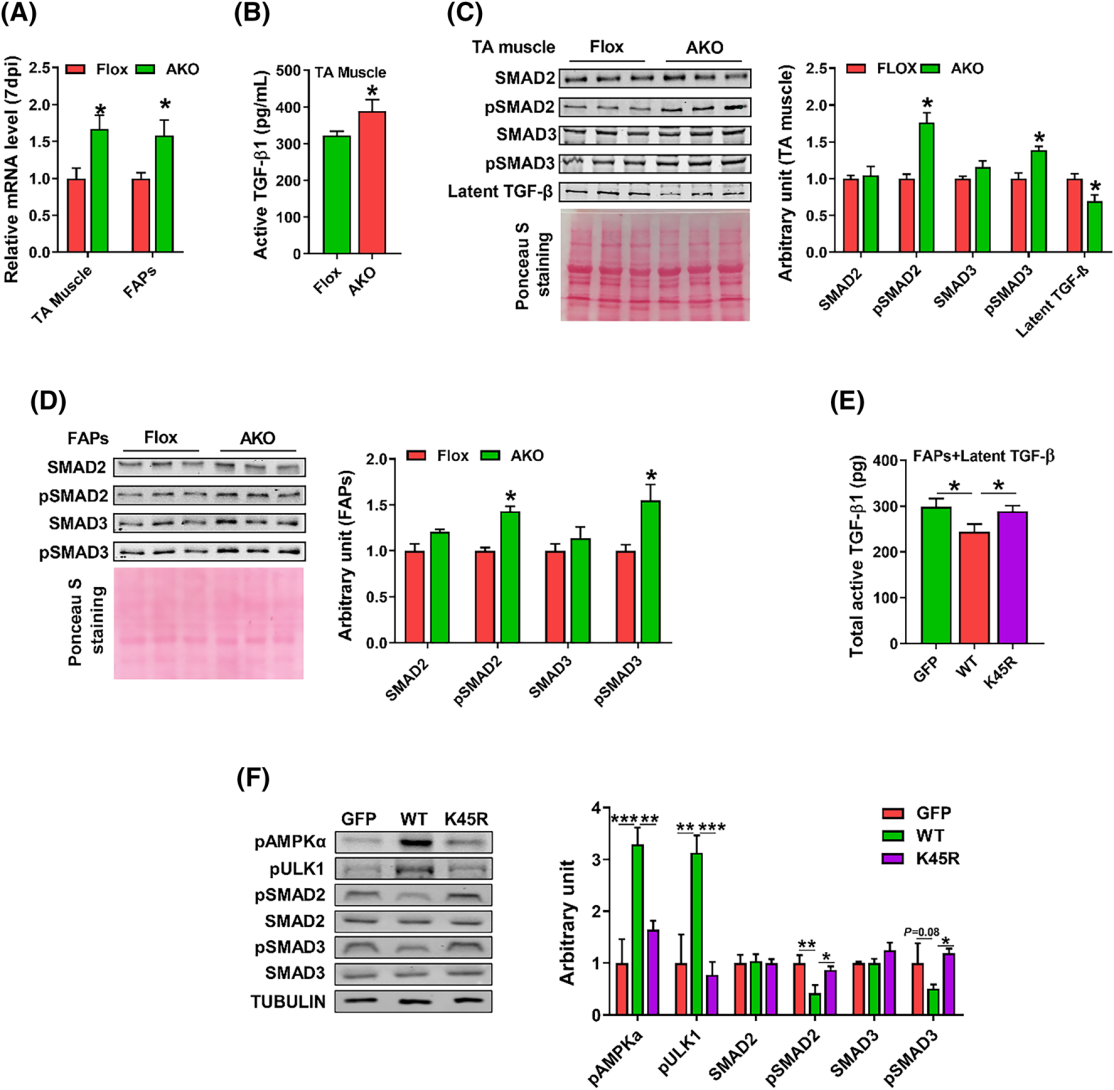
AMPKα1 knockout enhances transforming growth factor‐β (TGF‐β) signalling, which is associated with elevated fibrosis. (A) Relative mRNA expression of *Tgfb1* in fibro‐adipogenic progenitors (FAPs) and regenerating muscle at 7 days post‐injury (dpi). (B) The content of TGF‐β1 in tibialis anterior (TA) muscle at 7 dpi. (C) Western blotting analysis of latent TGF‐β, TGF‐β downstream mediators, including SMAD2, phospho (p)‐SMAD2, SMAD3 and p‐SMAD3 in regenerated muscle at 7 dpi. (D) Western blotting analysis of TGF‐β downstream mediators, including SMAD2, p‐SMAD2, SMAD3 and p‐SMAD3 in FAPs. (E) The total content of TGF‐β1 in the supernatant of culture media and FAPs, which were transfected with green fluorescent protein (GFP), wild‐type AMP‐activated protein kinase (AMPK) (WT) and K45R mutant AMPK (K45R); the culture media were supplemented with latent TGF‐β. (F) Western blotting analysis of phosphorylated AMPKα, a downstream target of AMPKα, phosphorylated ULK1 and TGF‐β downstream mediators in FAPs, which were transfected with GFP, WT and K45 mutant AMPK. Data are shown as the means ± SEM (*n* = 3). *P < 0.05, **P < 0.01, ***P < 0.001

AMPKα1 ablation increased TGF‐β mRNA expression in both TA muscle and FAPs (*Figure* *4* *A*). The content of active TGF‐β1 in TA muscle was measured by an ELISA kit. AMPKα1 KO increased the active TGF‐β1 content in the TA muscle (*Figure* *4* *B*). In addition, the AMPKα1 KO group had less latent TGF‐β, increased phospho‐SMAD2 and phospho‐SMAD3 contents, which are downstream mediators of TGF‐β signalling, but there was no difference in SMAD2 and SMAD3 contents between these two groups (*Figure* *4* *C*). We also analysed downstream mediators of TGF‐β in freshly isolated FAPs. Consistent with TA muscle, AMPK KO FAPs had higher contents of phospho‐SMAD2 and phospho‐SMAD3, but no difference was observed in SMAD2 and SMAD3 contents (*Figure* *4* *D*). To further confirm, we transferred FAPs with GFP, wild‐type AMPK (WT) and K45R mutant AMPK (K45R). Then obtained cells were cultured in serum‐free DMEM/F12 media supplemented with 2 ng/mL latent TGF‐β for 24 h. The active TGF‐β1 content was measured in cells and culture media supernatant. The K45R group had higher active TGF‐β1 than WT group (*Figure* *4* *E*). Moreover, the K45R group had lower levels of phospho‐AMPKα and phospho‐ULK1, which are the downstream mediators of AMPK signalling, compared with the WT group. AMPK mutation was associated with higher levels of pSMAD2 and pSMAD3, key signal transducers of TGF‐β signalling (*Figure* *4* *F*). These data revealed that AMPKα1 KO in FAPs enhanced activation of TGF‐β during muscle regeneration.

### AMPKα1 up‐regulates transforming growth factor‐β signalling via activating matrix metalloproteinase‐9

**Figure 5:**
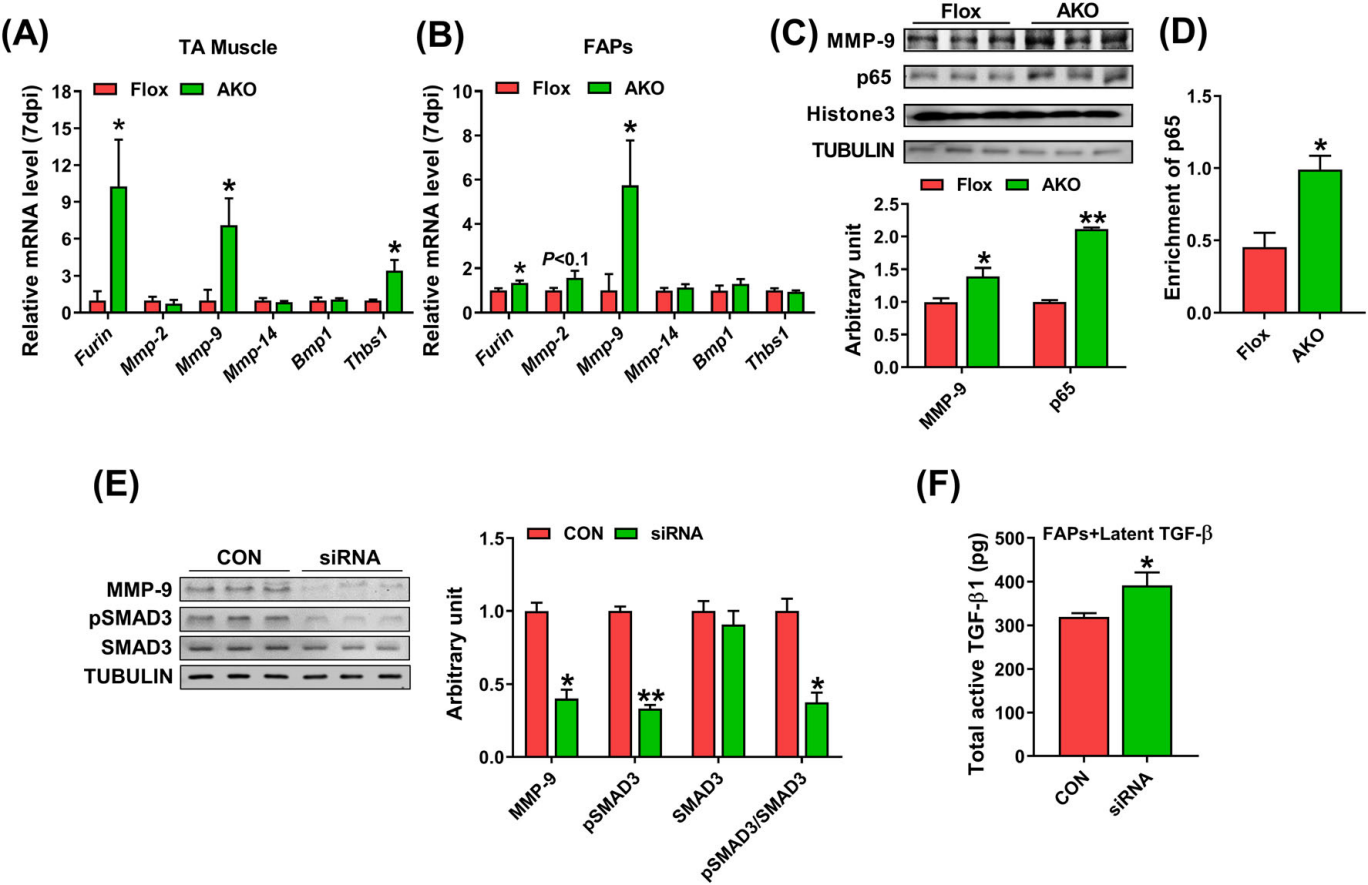
AMPKα1 regulates transforming growth factor‐β (TGF‐β) signalling via activating matrix metalloproteinase‐9 (MMP‐9). (A) Relative mRNA expression of enzyme activating TGF‐β in regenerating muscle at 7 days post‐injury (dpi), including *Furin*, *Mmp‐2*, *Mmp‐9*, *Mmp‐14*, *Bmp1* and *Thbs1*. (B) Relative mRNA expression of enzyme activating TGF‐β in fibro‐adipogenic progenitors (FAPs), including *Furin*, *Mmp‐2*, *Mmp‐9*, *Mmp‐14*, *Bmp1* and *Thbs1* in FAPs. (C) Western blotting analysis of MMP‐9 in FAPs and p65 in nucleus of FAPs. (D) Chromatin immunoprecipitation qPCR (ChIP‐qPCR) analysis of the enrichment of p65 in the promoter of *Mmp‐9*. (E) Western blotting analysis of MMP‐9 and TGF‐β downstream mediators, SMAD3 and phospho (p)‐SMAD3, in FAPs when MMP‐9 was knocked down. (F) The total content of TGF‐β1 in the supernatant of culture media and FAPs, in which MMP‐9 was knocked down and latent TGF‐β was supplemented. Data are shown as the means ± SEM (*n* = 3). *P < 0.05, **P < 0.01

After synthesis, cleavage is required to activate TGF‐β. We analysed the mRNA expression of enzymes regulating TGF‐β activity in TA muscle and FAPs at 7 dpi. In TA muscle, although AMPKα1 KO had no effect on the expression of *Mmp‐2*, *Mmp‐14* and *Bmp1*, AMPKα1 KO increased mRNA levels of *Furin*, *Mmp‐9* and *Thbs1* (*Figure* *5* *A*). In FAPs, *Furin* and *Mmp‐9* expression was increased by AMPKα1 KO, and there was an increased tendency of *Mmp‐2* expression in AMPKα1 KO FAPs (*Figure* *5* *B*). Interestingly, *Mmp‐9* expression was substantially enhanced by AMPKα1 KO in both TA muscle and FAPs. MMP‐9 is a member of the matrix metalloproteinases (MMPs) family, which can cleave and activate TGF‐β. MMP‐9 is regulated by NF‐κB signalling, which is inhibited by AMPK.^21^ ^, S5, S6^ To further explore the mechanism by which AMPK regulates MMP‐9 expression, we performed western blotting to measure p65 content in FAP nuclei via isolating subcellular fractionation and western blotting. We found that AMPKα1 KO promoted p65 translocation to nuclei (*Figure* *5* *C*). Also, using ChIP‐qPCR, we found that p65 binds to the *Mmp‐9* promoter, which was enhanced due to AMPKα1 KO (*Figure* *5* *D*).

To explore the direct effect of MMP‐9 on TGF‐β activation, MMP‐9 siRNA was used to knock down MMP‐9 in FAPs. Both CON and MMP‐9 knockdown groups were treated with latent TGF‐β for 2 days. Western blotting data showed that MMP‐9 content was decreased by siRNA treatment, which was associated with reduced phosphorylation of SMAD3, the downstream target of TGF‐β signalling (*Figure* *5* *E*). In addition, knockdown of MMP‐9 diminished the total content of active TGF‐β1 (*Figure* *5* *F*). These data show that AMPKα1 KO in FAPs elevates MMP‐9 expression and TGF‐β1 signalling.

### Obesity impairs muscle regeneration via inhibiting AMP‐activated protein kinase

**Figure 6:**
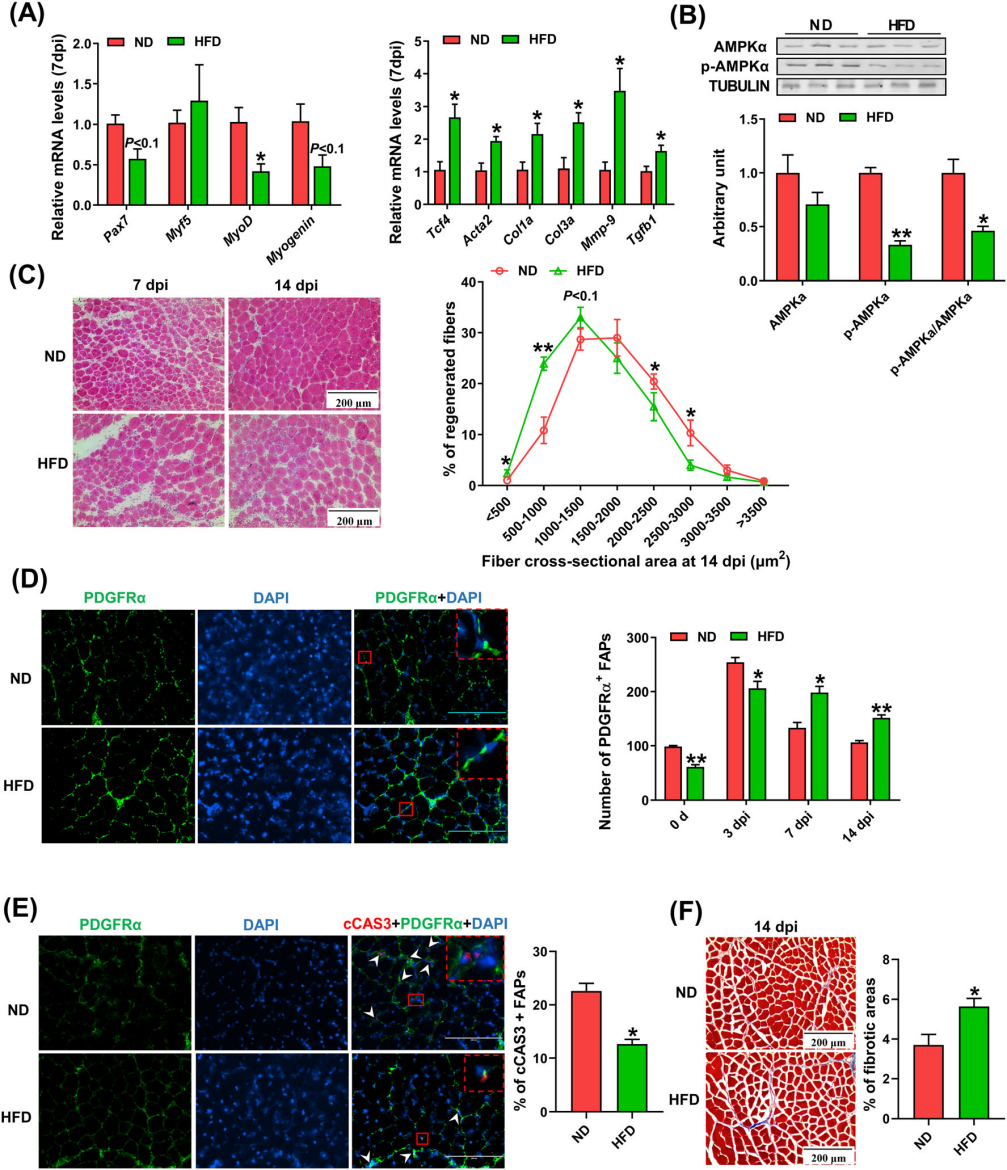
Obesity impairs muscle regeneration via suppressing AMP‐activated protein kinase (AMPK). C57BL/6J male mice were treated for 12 weeks of normal diet (ND) or high‐fat diet (HFD). (A) Relative mRNA expression of myogenic genes including *Pax7*, *Myf5*, *Myod* and *Myogenin*, and regulators involved in fibrosis including *Acta2*, *Tcf4*, *Col1a*, *Col3a*, *Tgfb1* and *Mmp‐9* at 7 days post‐injury (dpi) in tibialis anterior (TA) muscle. (B) Western blotting analysis of AMPKα and phospho (p)‐AMPKα in TA muscle at 7 dpi. (C) Haematoxylin and eosin (H&E) staining of TA muscle and distribution of cross‐sectional areas of regenerated myofibers (regenerated fibres with central nuclei). (D) Immunofluorescence staining of PDGFRα in regenerated TA muscle at 14 dpi and quantification of the number of PDGFRα+/DAPI+ fibro‐adipogenic progenitors (FAPs) per field at 3, 7 and 14 dpi. (E) Immunofluorescence analysis of cleaved Caspase 3 (cCAS3)+ FAPs and quantifications of the percentage of cCAS3+ FAPs. (F) Masson trichrome staining of regenerated muscle at 14 dpi and the percentage of connective tissue areas per field. Data are shown as the means ± SEM (*n* = 3). *P < 0.05, **P < 0.01. Bars, 200 μm

HFD treatment for 12 weeks significantly increased the body weight (*Figure* *S1* *A*), associated with higher fasting glucose and insulin levels, and elevated HOMA‐IR (*Figure* *S1* *B–D*). HFD group has higher content of active TGF‐β1 in TA muscle (*Figure* *S1* *E*). No significant difference in the ratio of muscle weight to tibia bone length was observed between lean and obese mice (*Figure* *S1* *F*). At 7 dpi, obesity attenuated mRNA expression of muscle regenerating markers, *MyoD*, and tended to decrease *Pax7* and *Myogenin* expression. The expression of fibrotic genes was enhanced in the obese group, including *Acta2*, *Tcf4*, *Col1α*, *Col3α* and *Tgfb1* (*Figure* *6* *A*). Compared with the lean mice, obese mice had lower phospho‐AMPKα content and the ratio of phospho‐AMPKα to AMPKα (*Figure* *6* *B*). As shown in H&E staining, the obese group had smaller regenerated muscle fibres compared with the lean group (*Figure* *6* *C*). The dynamic cellularity changes of FAPs were tracked by measuring PDGFRα+ cells at different stages, including 0 (before injury), 3, 7 and 14 dpi. Obesity impaired FAP proliferation at 0 and 3 dpi. Furthermore, the obesity group had more FAPs at 7 and 14 dpi, indicating that obesity enhanced apoptosis resistance of FAPs (*Figure* *6* *D*). Consistently, we observed lower cCAS3+ FAPs at 7 dpi (*Figure* *6* *E*) and more collagen accumulation at 14 dpi (*Figure* *6* *F*) in obese mice.

### The whole‐body AMPKα1 knockout in *R26CreER* ^*(+/+)*^ *Prkaa1* ^fl/fl^ mice impedes muscle regeneration with increased fibrosis

**Figure 7:**
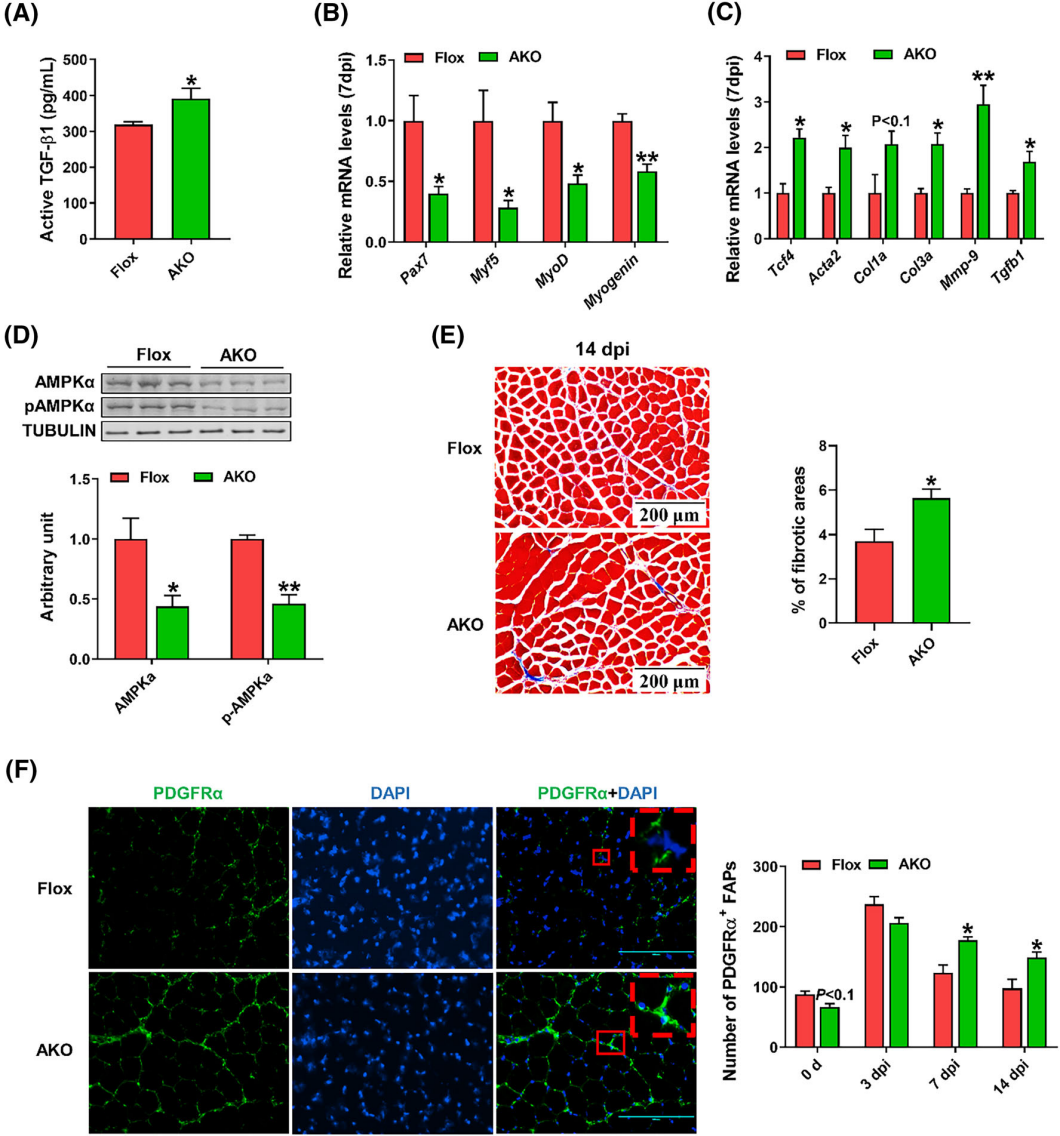
The whole‐body AMPKα1 knockout in *R26CreER* ^*(+/+)*^ *Prkaa1* ^fl/fl^ mice impedes muscle regeneration with increased fibrosis. *R26CreER* ^*(+/+)*^ *Prkaa1* ^fl/fl^ and *Prkaa1* ^fl/fl^ male mice were injected with tamoxifen. (A) The active transforming growth factor‐β1 (TGF‐β1) concentration in tibialis anterior (TA) muscle at 7 days post‐injury (dpi). (B) Relative mRNA expression of myogenic genes including *Pax7*, *Myf5*, *Myod* and *Myogenin* at 7 dpi. (C) Relative mRNA expression of regulators involved in fibrosis including *Acta2*, *Tcf4*, *Col1a*, *Col3a*, *Tgfb1* and *Mmp‐9* in 7 dpi TA muscle. (D) Western blotting analysis of AMPKα and phospho (p)‐AMPKα in TA muscle at 7 dpi. (E) Masson trichrome staining of regenerated muscle at 14 dpi and the percentage of connective tissue areas per field. (F) Immunofluorescence staining of PDGFRα in regenerated TA muscle at 14 dpi and quantification of the number of PDGFRα+/DAPI+ fibro‐adipogenic progenitors (FAPs) per field at 3, 7 and 14 dpi. Data are shown as the means ± SEM (*n* = 3). *P < 0.05, **P < 0.01. Bars, 200 μm

To mimic the whole‐body AMPK inhibition, we injected *R26CreER* ^*(+/+)*^ *Prkaa1* ^fl/fl^ and *Prkaa1* ^fl/fl^ mice with tamoxifen to induce the whole‐body AMPKα1 KO. At 7 dpi, the whole‐body AMPKα1 KO increased the content of active TGF‐β1 in TA muscle (*Figure* *7* *A*). The mRNA expression of muscle regenerating markers was down‐regulated in AMPKα1 KO group, including *Pax7*, *Myf5*, *MyoD* and *Myogenin* (*Figure* *7* *B*). In addition, the whole‐body AMPKα1 KO increased *Mmp‐9* and fibrogenic gene expression, including *Acta2*, *Tcf4*, *Col1α*, *Col3α* and *Tgfb1* (*Figure* *7* *C*). The western blotting showed that the content of AMPKα and phosphorylated AMPKα were decreased, showing that AMPKα was successfully knocked out (*Figure* *7* *D*). Masson trichrome staining was performed to evaluate the collagen deposition. Consistent with obese mice, the whole‐body AMPKα1 KO mice had higher content of collagen in TA muscle at 14 dpi (*Figure* *7* *E*). The dynamic population change of FAPs was measured using PDGFRα+ immunofluorescence staining at 0 (before injury), 3, 7 and 14 dpi. Loss of AMPKα1 tended to decrease FAPs at 0 dpi. However, at 7 and 14 dpi, AMPKα1 KO group had more FAPs than CON group (*Figure* *7* *F*). These data show that AMPK deficiency impairs muscle regeneration and induces muscle fibrosis through reducing FAP activation during the initial stage of muscle regeneration while suppressing their apoptosis during the resolution stage.

## Discussion

It has been established that FAPs are indispensable for muscle regeneration, and dysregulation of FAPs during muscle regeneration impairs muscle recovery from injury.^S7, S8^ FAPs secrete various molecules, including insulin‐like growth factor 1 (IGF‐1), follistatin and ILs, which promote satellite cell proliferation directly and indirectly.^10^, ^13^ ^, S9, S10^ In response to muscle injury, FAPs expand massively and peak at 3 dpi. Subsequently, pro‐inflammatory macrophages produce high amount of TNF‐α, which triggers FAP apoptosis and reduces FAP population to the pre‐damage level.^9^ ^, S11^ Prompt clearance of FAPs is necessary, because persistence of expanded FAPs can cause fibrotic and fatty infiltration.^15^ ^, S12^ In our study, AMPKα1 KO in FAPs and the whole body led to the persistence of FAPs at 7 and 14 dpi, accompanied by lower apoptosis. These results suggested that AMPKα1 KO elevated apoptosis resistance of FAPs at the recovery stage of muscle regeneration. Similarly, a recent study reported that exercise improves muscle regeneration through enhancing FAP apoptosis.^35^ Meanwhile, we found that AMPKα1 deletion promoted myofibroblast differentiation of FAPs, with higher expression of collagen genes and *Acta2* (encode αSMA, a marker protein of myofibroblasts). Myofibroblasts are one of the major sources of ECM, causing fibrosis.^S13^ Given that TGF‐β is a master fibrogenic factor,^S14, S15^ we explored the activation of TGF‐β during muscle generation.

TGF‐β is synthesized as a precursor protein, which needs to be cleaved to activate.^S16^ In the endoplasmic reticulum, a signal peptide, a prodomain (latency‐associated polypeptide [LAP]), a latent TGF‐β binding protein (LTBP) and the mature polypeptide form a complex. After the signal peptide is removed, this complex is called a large latency complex (LLC).^S17–S20^ In Trans‐Golgi, the LAP is cleaved but remains connected with the complex by non‐covalent bonds.^S21^ After secretion to the extracellular space, removal or proteolytic cleavage of LTBP and LAP is required to release and activate TGF‐β. MMP‐9 is a member of the MMP family, which can proteolytically cleave latent and release active TGF‐β.^S22, S23^ Our study showed that AMPKα1 KO increased mRNA and protein levels of MMP‐9 in FAPs, which is associated with reduction of the latent TGF‐β and increased active TGF‐β1 content. Consistently, it has been reported that pharmacological activation of AMPK suppresses mRNA expression of TGF‐β1.^9^ We observed deletion of AMPKα1 in FAPs enhanced TGF‐β1 mRNA level. To further verify the direct contribution of MMP‐9 to TGF‐β activation, the FAPs were transfected with siRNA to knock down MMP‐9 and treated with latent TGF‐β. The total active TGF‐β1 content was diminished in the MMP‐9 knockdown group, which was associated with decreased phosphorylated SMAD3, the downstream target of TGF‐β. These data demonstrated that AMPK regulates the activity of TGF‐β via MMP‐9.

To further explore how AMPK regulates MMP‐9, we examined NF‐κB signalling, which mediates MMP‐9 expression.^S23^ NF‐κB family has five subunits, which are separated into two groups. The first group includes RelA (p65), RelB and c‐Rel subunits. They have an N‐terminal Rel homology domain (RHD) and a C‐terminus transcriptional activation domain. The second group includes NF‐κB1 (p50) and NF‐κB2 (p52), comprising a N‐terminal RHD and a C‐terminal ankyrin repeat domain.^S24, S25^ Generally, κB family proteins are inhibited by binding to IκB proteins in the cytosol. After phosphorylation, IκB is ubiquitinated and degraded, releasing the NF‐κB and transferring into nuclei to regulate gene expression.^S26^ AMPK is a stronger inflammation suppressor, and previous studies reported that AMPK inhibits NF‐κB signalling pathway.^21^ ^, S5, S6^ AMPK activation attenuates NF‐κB translocation from the cytosol to the nucleus.^S27^ Moreover, AMPK activates other mediators to suppress NF‐κB signalling. For instance, AMPK activates SIRT1 deacetylase, which in turn suppresses NF‐κB signalling.^S28^ Additionally, AMPK enhances and activates peroxisome proliferator‐activated receptor‐gamma coactivator (PGC‐1α),^S29^ which in turn impairs NF‐κB activity.^S30–S32^ Our data revealed that impaired AMPKα1 in FAPs enhanced NF‐κB localization into nuclei; moreover, ChIP‐qPCR data showed that AMPKα1 KO enhanced the enrichment of p65 at the promoter of *Mmp‐9*, which explains the increased MMP‐9 expression.

In the past 50 years, the prevalence of obesity has increased profoundly and becomes a global pandemic.^S20^ Obesity is closely associated with serious health complications, including cardiovascular diseases, certain types of cancers, mental illness and type 2 diabetes.^S20^ Of note, obesity causes fibrotic accumulation and excessive fatty infiltration in regenerated muscle.^S33, S34^ In our study, we found that obesity decreased AMPK activity in the regenerating muscle. In addition, obese mice had enhanced *Mmp‐9* and *Tgfb1* expression. Obesity decreased the FAP population at 3 dpi and prevented FAPs from apoptosis at 7 and 14 dpi. Consistently, the obese group had less FAPs undergoing apoptosis at 7 dpi. At 14 dpi, we observed smaller regenerated muscle fibres and more collagen accumulation in obese mice. Obese subjects have reduced AMPK activity.^S35–S37^ To further confirm the role of AMPK inhibition under obese conditions, the *R26CreER* ^*(+/+)*^ *Prkaa1* ^fl/fl^ mice were used to knock out AMPKα1 in the whole body. Consistent with obese mice, AMPKα1 KO in the whole body led to collagen deposition at 14 dpi, associated with increased TGF‐β1 content at 7 dpi and FAPs at 7 and 14 dpi.

In conclusion, AMPK signalling has a potent role in the regulation of FAP apoptosis during muscle regeneration. Impaired AMPK increased p65 nuclear localization and MMP‐9 expression, which activated TGF‐β. Subsequently, enhanced activation of TGF‐β rendered apoptosis resistance and fibrotic differentiation of FAPs, elevating fibrosis in regenerated muscle. This study suggests that AMPK is a key therapeutic target to combat fibrosis during muscle regeneration, especially in obese patients.

## Conflict of interest

The authors declare no conflict of interest.

## Supporting information


**Figure S1.** AMPKα1 knockout promotes fibrosis in regenerated muscle tissue. A, Relative mRNA expression of fibrotic markers and TGF‐β downstream targets including *Acta2*, *Col1a*, *Col3a*, *EDA‐fibronectin*, *Fibronectin* and Tcf4 in regenerated muscle at 7 days post‐injury (dpi). B, Immunofluorescence analysis of satellite cells in muscle at 3 and 7 dpi. C, Procaspase3 and cleaved caspase3 in FAPs. *P < 0.05. **P < 0.01. Bars, 100 μm.
**Figure S2.** Obesity impairs muscle regeneration via suppressing AMPK. C57BL/6 J male mice were treated for 12 weeks of normal diet (ND) or high fat diet (HFD). A, Body weight. B ‐ D, Blood glucose (B), insulin (C) and calculated HOMA‐IR (D) after 5 h fasting. E, The active TGF‐β1 level in tibialis anterior (TA) muscle at post‐injury (dpi). F, The weight of TA muscle normalized to tibia bone length at 3, 7 and 14 dpi. G, Immunofluorescence analysis of TCF4 labeled fibroblasts at 7 dpi. *P < 0.05. **P < 0.01. Bars, 100 μm.
**Figure S3.** The whole body AMPKα1 knockout in R26CreER(+/+) Prkaa1fl/fl mice had increased TCF4+ fibroblasts at 7 post‐injury (dpi). **P < 0.01. Bars, 100 μm.
