# Supplementary material for: AMP‐activated protein kinase inhibition in fibro‐adipogenic progenitors impairs muscle regeneration and increases fibrosis
Source: J Cachexia Sarcopenia Muscle. 2022 Dec 13;14(1):479–92. doi: 10.1002/jcsm.13150 (PMC9891933; doi:10.1002/jcsm.13150)
Supplement: Supplementary file 1 — Figure S1. AMPKα1 knockout promotes fibrosis in regenerated muscle tissue. A, Relative mRNA expression of fibrotic markers and TGF‐β downstream targets including Acta2, Col1a, Col3a, EDA‐fibronectin, Fibronectin and Tcf4 in regenerated muscle at 7 days post‐injury (dpi). B, Immunofluorescence analysis of satellite cells in muscle at 3 and 7 dpi. C, Procaspase3 and cleaved caspase3 in FAPs. *P < 0.05. **P < 0.01. Bars, 100 μm. Figure S2. Obesity impairs muscle regeneration via suppressing AMPK. C57BL/6 J male mice were treated for 12 weeks of normal diet (ND) or high fat diet (HFD). A, Body weight. B ‐ D, Blood glucose (B), insulin (C) and calculated HOMA‐IR (D) after 5 h fasting. E, The active TGF‐β1 level in tibialis anterior (TA) muscle at post‐injury (dpi). F, The weight of TA muscle normalized to tibia bone length at 3, 7 and 14 dpi. G, Immunofluorescence analysis of TCF4 labeled fibroblasts at 7 dpi. *P < 0.05. **P < 0.01. Bars, 100 μm. Figure S3. The whole body AMPKα1 knockout in R26CreER(+/+) Prkaa1fl/fl mice had increased TCF4+ fibroblasts at 7 post‐injury (dpi). **P < 0.01. Bars, 100 μm. [file JCSM-14-479-s001.pdf]

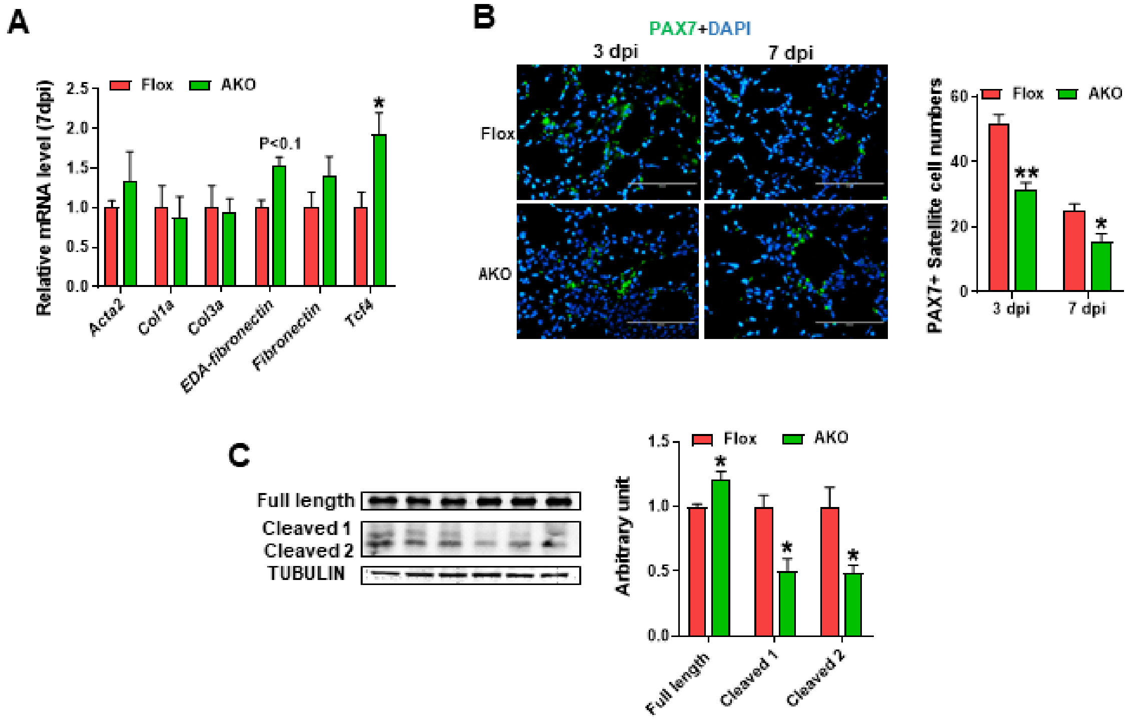

Supplementary figure 1. AMPKα1 knockout promotes fibrosis in regenerated muscle tissue. A, Relative mRNA expression of fibrotic markers and TGF-β downstream targets including *Acta2*, *Col1a*, *Col3a*, *EDA-fibronectin*, *Fibronectin* and Tcf4 in regenerated muscle at 7 days post-injury (dpi). B, Immunofluorescence analysis of satellite cells in muscle at 3 and 7 dpi. C, Procaspase3 and cleaved caspase3 in FAPs. $*P < 0.05$. $**P < 0.01$. Bars, 100 μm.

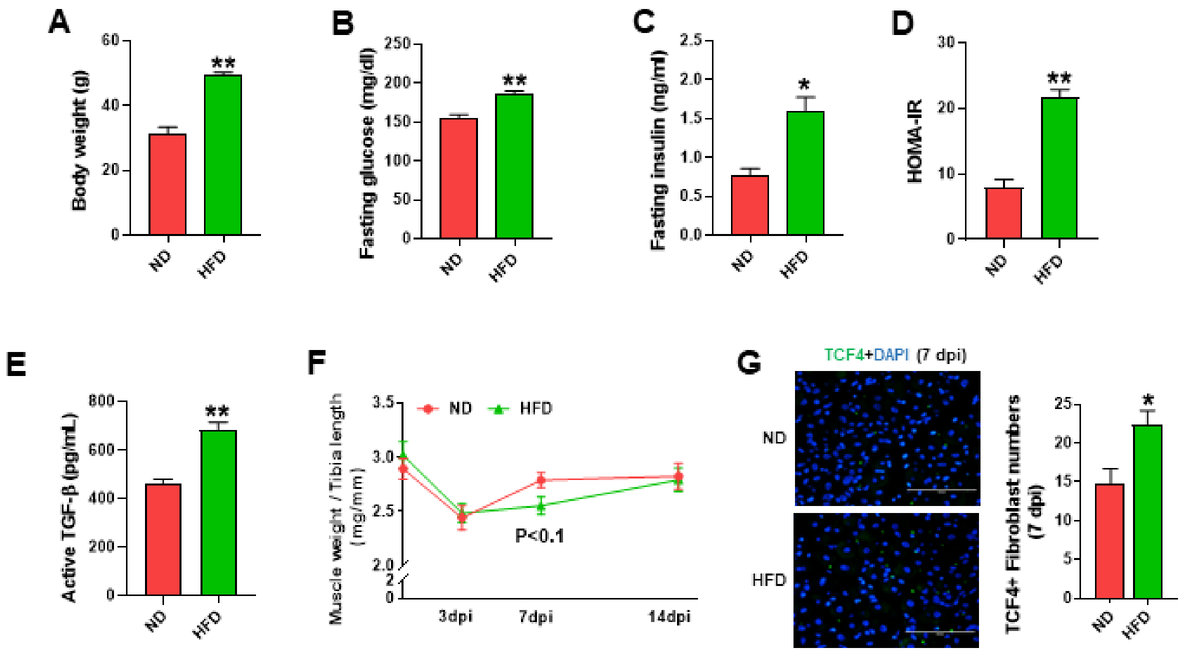

Supplementary figure 2. Obesity impairs muscle regeneration via suppressing AMPK. C57BL/6J male mice were treated for 12 weeks of normal diet (ND) or high fat diet (HFD). A, Body weight. B - D, Blood glucose (B), insulin (C) and calculated HOMA-IR (D) after 5 h fasting. E, The active TGF-β1 level in tibialis anterior (TA) muscle at post-injury (dpi). F, The weight of TA muscle normalized to tibia bone length at 3, 7 and 14 dpi. G, Immunofluorescence analysis of TCF4 labeled fibroblasts at 7 dpi. $*P < 0.05$. $**P < 0.01$. Bars, 100 μm.

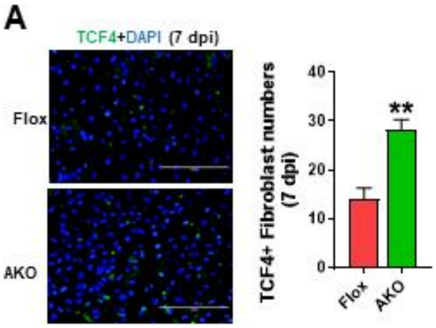

Supplementary figure 3. The whole body AMPKα1 knockout in R26CreER(+/+) Prkaa1fl/fl mice had increased TCF4+ fibroblasts at 7 post-injury (dpi). $**P < 0.01$. Bars, 100 μm.

References

S1. Jiang S, Li T, Yang Z, Yi W, Di S, Sun Y, et al. AMPK orchestrates an elaborate cascade protecting tissue from fibrosis and aging. Ageing Res Rev. 2017;38:18-27.

S2. Murphy MM, Lawson JA, Mathew SJ, Hutcheson DA, Kardon G. Satellite cells, connective tissue fibroblasts and their interactions are crucial for muscle regeneration. Development. 2011;138:3625-37.

S3. Guiraud S, Edwards B, Squire SE, Moir L, Berg A, Babbs A, et al. Embryonic myosin is a regeneration marker to monitor utrophin-based therapies for DMD. Hum Mol Genet. 2019;28:307-19.

S4. Schiaffino S, Gorza L, Sartore S, Saggin L, Carli M. Embryonic myosin heavy chain as a differentiation marker of developing human skeletal muscle and rhabdomyosarcoma. A monoclonal antibody study. Experimental cell research. 1986;163:211-20.

S5. Hattori Y, Suzuki K, Hattori S, Kasai K. Metformin inhibits cytokine-induced nuclear factor kappaB activation via AMP-activated protein kinase activation in vascular endothelial cells. Hypertension. 2006;47:1183-8.

S6. Xiang HC, Lin LX, Hu XF, Zhu H, Li HP, Zhang RY, et al. AMPK activation attenuates inflammatory pain through inhibiting NF-kappaB activation and IL-1beta expression. J Neuroinflammation. 2019;16:34.

S7. Lukjanenko L, Karaz S, Stuelsatz P, Gurriaran-Rodriguez U, Michaud J, Dammone G, et al. Aging Disrupts Muscle Stem Cell Function by Impairing Matricellular WISP1 Secretion from Fibro-Adipogenic Progenitors. Cell Stem Cell. 2019;24:433-46 e7.

S8. Mozzetta C, Consalvi S, Saccone V, Tierney M, Diamantini A, Mitchell KJ, et al. Fibroadipogenic progenitors mediate the ability of HDAC inhibitors to promote regeneration in dystrophic muscles of young, but not old Mdx mice. EMBO Mol Med. 2013;5:626-39.

S9. Madaro L, Passafaro M, Sala D, Etxaniz U, Lugarini F, Proietti D, et al. Denervation-activated STAT3-IL-6 signalling in fibro-adipogenic progenitors promotes myofibres atrophy and fibrosis. Nature cell biology. 2018;20:917-27.

S10. Formicola L, Pannerec A, Correra RM, Gayraud-Morel B, Ollitrault D, Besson V, et al. Inhibition of the Activin Receptor Type-2B Pathway Restores Regenerative Capacity in Satellite Cell-Depleted Skeletal Muscle. Front Physiol. 2018;9:515.

S11. Davies MR, Liu X, Lee L, Laron D, Ning AY, Kim HT, et al. TGF-beta Small Molecule Inhibitor SB431542 Reduces Rotator Cuff Muscle Fibrosis and Fatty Infiltration By Promoting Fibro/Adipogenic Progenitor Apoptosis. PloS one. 2016;11:e0155486.

S12. Giuliani G, Rosina M, Reggio A. Signaling pathways regulating the fate of fibro/adipogenic progenitors (FAPs) in skeletal muscle regeneration and disease. FEBS J. 2021.

S13. Klingberg F, Hinz B, White ES. The myofibroblast matrix: implications for tissue repair and fibrosis. J Pathol. 2013;229:298-309.

S14. Derynck R, Budi EH. Specificity, versatility, and control of TGF-beta family signaling. Sci Signal. 2019;12.

S15. Rifkin DB. Latent transforming growth factor-beta (TGF-beta) binding proteins: orchestrators of TGF-beta availability. The Journal of biological chemistry. 2005;280:7409-12.

S16. Khalil N. TGF-beta: from latent to active. Microbes Infect. 1999;1:1255-63.

S17. Luppino FS, de Wit LM, Bouvy PF, Stijnen T, Cuijpers P, Penninx BW, et al. Overweight, obesity, and depression: a systematic review and meta-analysis of longitudinal studies. Arch Gen Psychiatry. 2010;67:220-9.

S18. Lega IC, Lipscombe LL. Review: Diabetes, Obesity, and Cancer-Pathophysiology and Clinical Implications. Endocr Rev. 2020;41.

S19. Koliaki C, Liatis S, Kokkinos A. Obesity and cardiovascular disease: revisiting an old relationship. Metabolism: clinical and experimental. 2019;92:98-107.

S20. Bluher M. Obesity: global epidemiology and pathogenesis. Nat Rev Endocrinol. 2019;15:288-98.

S21. Distler JHW, Gyorfi AH, Ramanujam M, Whitfield ML, Konigshoff M, Lafyatis R. Shared and distinct mechanisms of fibrosis. Nat Rev Rheumatol. 2019;15:705-30.

S22. Kobayashi T, Kim H, Liu X, Sugiura H, Kohyama T, Fang Q, et al. Matrix metalloproteinase-9 activates TGF-beta and stimulates fibroblast contraction of collagen gels. Am J Physiol Lung Cell Mol Physiol. 2014;306:L1006-15.

S23. Yu Q, Stamenkovic I. Cell surface-localized matrix metalloproteinase-9 proteolytically activates TGF-beta and promotes tumor invasion and angiogenesis. Genes & development. 2000;14:163-76.

S24. Perkins ND. The diverse and complex roles of NF-kappaB subunits in cancer. Nat Rev Cancer. 2012;12:121-32.

S25. Li H, Malhotra S, Kumar A. Nuclear factor-kappa B signaling in skeletal muscle atrophy. Journal of molecular medicine. 2008;86:1113-26.

S26. Oeckinghaus A, Ghosh S. The NF-kappaB family of transcription factors and its regulation. Cold Spring Harbor perspectives in biology. 2009;1:a000034.

S27. Murphy-Ullrich JE, Poczatek M. Activation of latent TGF-beta by thrombospondin-1: mechanisms and physiology. Cytokine Growth Factor Rev. 2000;11:59-69.

S28. Yeung F, Hoberg JE, Ramsey CS, Keller MD, Jones DR, Frye RA, et al. Modulation of NF-kappaB-dependent transcription and cell survival by the SIRT1 deacetylase. The EMBO journal. 2004;23:2369-80.

S29. Jager S, Handschin C, St-Pierre J, Spiegelman BM. AMP-activated protein kinase (AMPK) action in skeletal muscle via direct phosphorylation of PGC-1alpha. Proceedings of the National Academy of Sciences of the United States of America. 2007;104:12017-22.

S30. Hah N, Lee ST. An absolute role of the PKC-dependent NF-kappaB activation for induction of MMP-9 in hepatocellular carcinoma cells. Biochemical and biophysical research communications. 2003;305:428-33.

S31. Shin Y, Yoon SH, Choe EY, Cho SH, Woo CH, Rho JY, et al. PMA-induced up-regulation of MMP-9 is regulated by a PKCalpha-NF-kappaB cascade in human lung epithelial cells. Exp Mol Med. 2007;39:97-105.

S32. Morizane Y, Thanos A, Takeuchi K, Murakami Y, Kayama M, Trichonas G, et al. AMP-activated protein kinase suppresses matrix metalloproteinase-9 expression in mouse embryonic fibroblasts. The Journal of biological chemistry. 2011;286:16030-8.

S33. Hu Z, Wang H, Lee IH, Modi S, Wang X, Du J, et al. PTEN inhibition improves muscle regeneration in mice fed a high-fat diet. Diabetes. 2010;59:1312-20.

S34. Mogi M, Kohara K, Nakaoka H, Kan-No H, Tsukuda K, Wang XL, et al. Diabetic mice exhibited a peculiar alteration in body composition with exaggerated ectopic fat deposition after muscle injury due to anomalous cell differentiation. J Cachexia Sarcopenia Muscle. 2016;7:213-24.

S35. Xu XJ, Gauthier MS, Hess DT, Apovian CM, Cacicedo JM, Gokce N, et al. Insulin sensitive and resistant obesity in humans: AMPK activity, oxidative stress, and depot-specific changes in gene expression in adipose tissue. Journal of lipid research. 2012;53:792-801.

S36. Martin TL, Alquier T, Asakura K, Furukawa N, Preitner F, Kahn BB. Diet-induced obesity alters AMP kinase activity in hypothalamus and skeletal muscle. The Journal of biological chemistry. 2006;281:18933-41.

S37. Jung Y, Park J, Kim HL, Sim JE, Youn DH, Kang J, et al. Vanillic acid attenuates obesity via activation of the AMPK pathway and thermogenic factors in vivo and in vitro. FASEB J. 2018;32:1388-402.

S38. von Haehling S, Morley JE, Coats AJS, Anker SD. Ethical guidelines for publishing in the Journal of Cachexia, Sarcopenia and Muscle: update 2017. J Cachexia Sarcopenia Muscle 2017;8: 1081-1083
